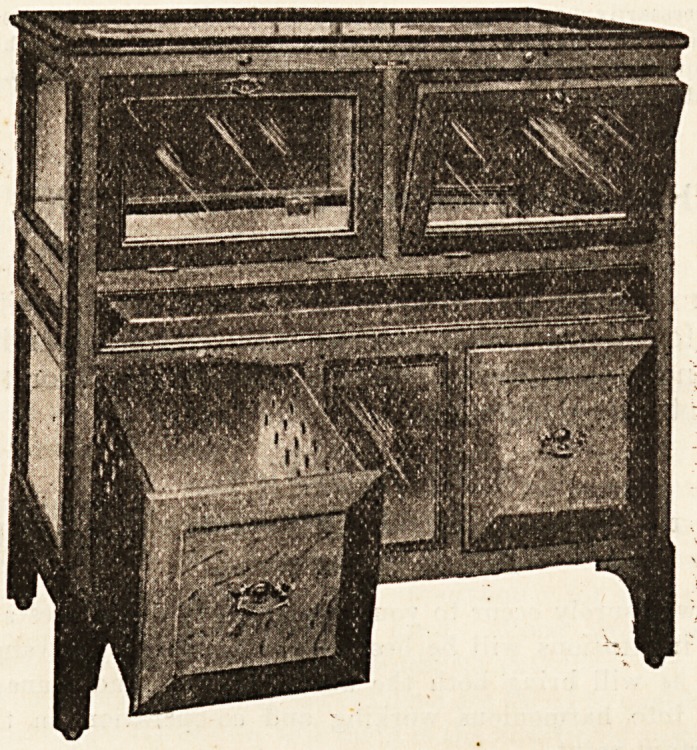# New Appliances & Things Medical

**Published:** 1910-04-09

**Authors:** 


### NEW APPLIANCES & THINGS MEDICAL.

[We shall be glad to receive at our Office, 28 & 29 Southampton Street, Strand, London, W.C., from the manufacturers, specimens of all new preparations and appliances.]

### A USEFUL BED-PAN.

(The Meinecke "Perfection" Bed and Douche Pan. Grimwades, Stoke-on-Trent.)

There is no appliance more commonly used in hospitals than the bed-pan, and yet it is strange how little care is given to its selection. In many instances hospital superintendents are inclined to buy the cheapest pan without regard to the comfort of the patient or the work of the nurse, or it not infrequently happens that the superintendent pays so little attention to the matter that any bed-pan is bought. Great strides have been made in recent years in the art of providing for the comfort of the patient and lessening the work of the nurse, and in no direction has this been more evident than in the making of hospital bed-pans. In the opinion of those competent to judge, one of the best models obtainable to-day is the Meinecke "Perfection" combined bed and douche pan. This bed-pan is in use in many of the great hospitals throughout the country, where its merits have been quickly recognised. The "Perfection" bed-pan has been designed with two distinct objects: First, to give comfort to the patient by making a pan which fits the shape of the body, and therefore adds greatly to the comfort of the patient; and second, to save work for the nurse by providing an article which is easily and quickly emptied and cleansed. Hospital superintendents who have not seen this pan will find it to their advantage to inspect it and give it a trial in their institutions. The "Perfection" bad-pan, which can be obtained from all wholesale chemists and hospital supply houses, is manufactured by Grimwades, Ltd., Stoke-on-Trent.

### TWYFORDS' SANITARY APPLIANCES.

(Twyfords, Limited, Cliffe Vale Potteries and Enamelled Fireclay Works, Hanley, Staffordshire.)

The appliances and fixtures made by this firm are all excellent models which have been thoroughly tested in various institutions and which have given universal satisfaction wherever they have been employed. The firm publishes a useful portfolio catalogue which gives full details of the chief specialities. We would draw special attention to the mortuary table listed in this catalogue. It is a portable model in white enamel "adamant" fireclay—a glazed surface that stands hard wear and the strenuous usage of the post-mortem room very well —built on a carriage frame of galvanised wrought iron with rubber-tyred wheels; it is one of the simplest and at the same time one of the most useful models for institutions. Another very excellent model is the portable bath, which is of enamelled copper with an easily moved carriage on swivel bearings. The lavatory appliances supplied by Twyfords are nearly all in "Adamant" fireclay, a clay compound which has been subjected to a very high temperature and has been made extremely durable, with a highly glazed pure white vitreous surface. This glaze does not easily chip and wears well, with the result that the appliances always look neat and new, since they are easily cleansed. Institutional workers who are not yet acquainted with the Twyford specialities should lose no time in sending for the illustrated catalogue and testing for themselves the value of the appliances manufactured by this firm.

### THE NEW TELESCOPIC SPLINT.

(Medical Requisites Co., 54 Deansgate, Manchester.)

This splint has received honourable mention from orthopædists who have tried it, and anyone who has used it will be reluctant to go back to the old-fashioned, cumbrous Thomas, however useful the latter may be. In the case of Slater's telescopic hip and knee splints the adjustments are so easy and the application so simple that the temptation to leave the selection and fitting of the splint to the makers is very great. We do not wish to be misunderstood. The splints are excellent, but they should only be prescribed by a medical man, and the prescriber should satisfy himself that the particular case is one in which this variety of splint is likely to do the most good. The price of the instrument is not stated.

### PARKER'S PATENT CRYSTAL COOLERS.

(Parker Bros., 104, 106, 108, 110 Curtain Road and Bateman's Row, London, E.C.)

The importance of refrigerating and cooling apparatus to institutions need no longer be insisted upon; every institutional worker has recognised that a hospital without cooling apparatus is but half a hospital, considered from a modern point of view. The only difference of opinion is as to the particular models which give most satisfaction. It may at once be stated that the secretary or superintendent who experiments with cheap models is likely to regret his rashness. A good cooler is the result of careful testing and prolonged experiment, and it is fortunate for hospital workers that these experiments are usually done by the most exacting of men, hotel chefs. A cooler that has stood the hotel test is one, therefore, that can be safety recmmended fr institutional trial. All the models stocked by Messrs. Parker have stood this test. They are neatly constructed, airy and attractive looking double glass houses, with an insulating air space. Their use means a great economy in ice consumption. With a consumption of fourteen pounds of ice per day the temperature averages fourteen degrees lower within the cooler than in the room where the glass chamber is kept. For hospital and institutional use the No. 100 model is recommended. This model, of which we append an illustration, is now used in many large hospitals, infirmaries, and hotels, and has everywhere given complete satisfaction. It is cheap in price, and, like all the other coolers supplied by Parker Bros., durable and attractive in construction. For doctors who wish to have a cold serum chest in their consulting room the smaller models—for example No. ?, (which is listed at £8 10s.)—are to be preferred. The firm supplies a useful catalogue which gives full particulars regarding these coolers.

## Figures and Tables

**Figure f1:**